# A Validation of the French Version of the Attitudes to Aging Questionnaire (AAQ): Factor Structure, Reliability and Validity

**DOI:** 10.5334/pb.301

**Published:** 2016-03-22

**Authors:** Manon Marquet, Pierre Missotten, Sarah Schroyen, Iris van Sambeek, Marjan van den Akker, Carine Van Den Broeke, Frank Buntinx, Stéphane Adam

**Affiliations:** 1 University of Liège, BE; 2 Maastricht University, NL; 3 KU Leuven, BE

**Keywords:** Attitudes to Aging Questionnaire (AAQ), aging, factor structure, reliability, validity

## Abstract

**Introduction:** The Attitudes to Aging Questionnaire (AAQ) was developed to measure attitudes toward the aging process as a personal experience from the perspective of older people. The present study aimed to validate the French version of the AAQ.

**Participants and methods:** This study examined factor structure, acceptability, reliability and validity of the AAQ’s French version in 238 Belgian adults aged 60 years or older. In addition, participants provided information on demographics, self-perception of their mental and physical health (single items), quality of life (WHOQOL-OLD) and social desirability (DS-36).

**Results:** Exploratory Factor Analysis produced a three-factor solution accounting for 36.9% of the variance. No floor or ceiling effects were found. The internal consistency, measured by Cronbach’s alpha coefficients for the AAQ subscales were 0.62 (Physical Change), 0.74 (Psychological Growth), and 0.75 (Psychosocial Loss). A priori expected associations were found between AAQ subscales, self-reported health and quality of life, indicating good convergent validity. The scale also showed a good ability to discriminate between people with lower and higher education levels, supporting adequate known-groups validity. Finally, we confirmed the need to control for social desirability biases when assessing self-reported attitudes toward one’s own aging.

**Conclusion:** The data support the usefulness of the French version of the AAQ for the assessment of attitudes toward their own aging in older people.

## Introduction

Since the older population has grown faster than the total population, the proportion of older persons relative to the rest of the population has considerably increased: 9.2% of the world’s population was at least 60 years of age in 1950; this number has now risen to 11.7% in 2013 and it is projected to reach 21.1% in 2050 (United Nations, 2013). In Western societies, this increasing proportion of older people is associated with a view of aging which is deeply focused on decline. Particularly, even if older people are perceived as warm by younger individuals, they are often considered as physically and cognitively inept (Cuddy & Fiske, 2002; Cuddy et al., 2009). These essentially negative stereotypes allude to the concept of “ageism” (Butler, 1969), which refers to negative attitudes toward the elderly.

According to the Levy’s stereotype embodiment theory (Levy, 2009), prolonged exposure to negative aging stereotypes during childhood and adulthood results in people developing negative stereotypes about the older population. This phenomenon has consequences: a long-term follow-up study among 395 people aged 22 years at baseline showed that people with more negative age stereotypes (at baseline) demonstrated significantly worse memory performance over 38 years than those with less negative age stereotypes (Levy, Zonderman, Slade, & Ferrucci, 2012). More interestingly, memory decline was significantly greater among those who had reached or passed the age at which they considered someone old. Similarly, a study showed that among participants aged 49 and younger, those who held negative age stereotypes were significantly more likely to experience a cardiovascular event in the following 38 years than those with more positive age stereotypes (Levy, Zonderman, Slade, & Ferrucci, 2009).

These studies illustrate the fact that as people grow older and reach old age, negative age stereotypes become self-relevant. In other words, people progressively become the target of the aging stereotypes they have assimilated from their surrounding social environment during past decades, which may shape their attitudes toward their own aging when they view themselves as old (Levy, 2009). Attitudes toward one’s own aging include affective, cognitive and evaluative components of behavior regarding the process of aging as a personal experience (Hess, 2006). Recent cross-sectional studies have demonstrated that negative attitudes toward one’s own aging, measured by the Attitudes to Aging Questionnaire, (AAQ; Laidlaw, Power, & Schmidt, 2007), are strongly associated with lower quality of life (Top & Dikmetas, 2012; Top, Eris, & Kabalcioglu, 2012a, 2012b). In addition, several longitudinal studies have shown that attitudes toward one’s own aging, usually assessed by the Attitude toward own aging subscale (ATOA) of the Philadelphia Geriatric Moral Scale (PGCMS; Lawton, 1975) can have either deleterious or beneficial long-term effects on various health outcomes, depending on their negative or positive valence. In studies involving older adults, Levy and colleagues showed that people with negative attitudes at baseline describe themselves as having worse physical health over a 28-year period (Levy, Slade, & Kasl, 2002) and live an average of 7.5 years shorter than those with more positive attitudes (Levy, Slade, Kunkel, & Kasl, 2002). One explanation suggests that these deleterious effects of negative attitudes are related to daily attitudes in life: people with a negative view of their own aging were less likely to engage in good health behaviors (e.g., healthy diet, use of seatbelts, physical exercise, minimization of alcohol or tobacco consumption) over the course of 20 years, during which they were followed up (Levy & Myers, 2004).

These results highlight the necessity to take into account age stereotypes held by older people, and particularly their attitudes toward their own aging rather than objective indicators of physical or psychological distress. Moreover, it is important not to neglect that attitudes toward one’s own aging is only one expression of subjective aging (Diehl et al., 2014). In fact, subjective aging is a superordinate construct encompassing several related concepts reflecting the ways in which individuals experience the aging process (Brothers, Miche, Wahl, & Diehl, 2015). For example, another expression of subjective aging is the subjective age which refers to the age an individual feels like or views himself (Diehl et al., 2014). On this subject, it has already been demonstrated that feeling younger is associated with more positive attitudes toward one’s own aging (Brothers et al., 2015).

Even if there has been a growing interest in studying the aging process as a personal experience and attitudes toward it during the last decade, there is a remarkable lack of instruments to assess these variables. In long-term follow-up studies conducted by Levy and colleagues, the ATOA was used to assess attitudes toward one’s own aging. This subscale comprises five items (statements) used to capture the general attitude with which an individual approaches his or her own aging (e.g., for the statement “Things keep getting worse as I get older”, the respondent is asked to indicate if he agrees or disagrees). Thus, the ATOA seems inadequate as a general tool to flexibly and comprehensively measure attitudes toward one’s own aging (Laidlaw et al., 2007).

In this context, the World Health Organization Quality of Life (WHOQOL) Group developed in 2007 the Attitudes to Aging Questionnaire (Laidlaw et al., 2007), an outcome which is better suited to flexibly and comprehensively assess attitudes toward the aging process as a personal experience from the perspective of older adults. Even though both the ATOA and the AAQ take into account the losses and gains experienced over the life course, the AAQ has the following advantages: its conceptualization of older adults’ attitudes toward their own aging is multidimensional and it can be applied in cross-cultural settings. Indeed, this original self-report instrument was based on an intense theoretical debate among international experts, which proposed the instrument’s items; these were later tested in focus groups in 15 countries, carried out with older adults to confirm or adjust their content. Such perspective is rare, but important, because older people are the only part of society qualified to comment on the experience of aging: they have the most intimate knowledge of adaptation to the aging process (Lucas-Carrasco, Laidlaw, Gomez-Benito, & Power, 2013). The AAQ primarily focuses on three different aspects of aging. The first subscale focuses on Psychosocial Losses and includes items related to negative attitudes toward own aging. For adults experiencing psychosocial losses, old age is perceived as a negative experience combining psychological and social losses (e.g., “As I get older I find it more difficult to make new friends”). The second subscale (Physical Change) focuses on physical functioning (health and exercise) and the experience of aging itself. It relies on a positive view of aging and physical functioning (e.g., “I have more energy now than I expected for my age”). The third subscale is entitled “Psychological Growth”. Feelings of growth indicate that aging is perceived as a time associated with wisdom and growth. The lifespan development perspective on aging is therefore particularly explicit in this subscale (e.g., “Wisdom comes with age”) which also highlights gains for oneself and for others (e.g., “I want to give a good example to younger people”). Concerning the format of the AAQ, it utilizes a combination of the general and experiential approach to aging. For example, the general approach comprises items such as “Old age is a time of loneliness” and the experiential approach includes items such as “I see old age mainly as a time of loss”. Note that even if the AAQ utilizes both an experiential and general approach, the questionnaire’s instructions specify that, in their answers, people need to report how they feel about growing older and to consider their life in general. Therefore, both the general and experiential approaches are intended to reflect older people’s attitudes toward their own aging.

The validity and reliability of the AAQ have been tested in an international dataset (Laidlaw et al., 2007). Validation studies have also been conducted in Spanish (Lucas-Carrasco et al., 2013), Brazilian (Chachamovich, Fleck, Trentini, Laidlaw, & Power, 2008), Scottish (Shenkin, Watson, Laidlaw, Starr, & Deary, 2014), Norwegian and English Canadian populations (Kalfoss, Low, & Molzahn, 2010). These studies demonstrated that the questionnaire had good psychometric properties. Particularly, they showed that when older participants had positive attitudes toward their own aging, they felt in good health and the better was their quality of life (Kalfoss et al., 2010, Lucas Carrasco et al., 2013). People with more positive attitudes also reported less depressive symptoms (Chachamovich et al., 2008; Kalfoss et al., 2010, Lucas-Carrasco et al., 2013). Moreover, it seems that even if attitudes toward one’s own aging are not influenced by participant’s gender, they differ according to the level of education, with higher educated people reporting more positive attitudes (Lucas-Carrasco et al., 2013). Nevertheless, according to the results of the analyses aimed at confirming the three dimensions of the AAQ (Chachamovich, et al., 2008; Kalfoss, Low, & Molzahn, 2010, Laidlaw et al., 2007; Skenkin et al., 2014), it appears that the factor structure of the AAQ is not as clear as one might expect (e.g., item cross-loadings). Therefore, this study aimed at contributing to the existing literature by exploring the dimensions of the French adaptation of the AAQ and examining its validity and reliability. Moreover, the availability of such a tool in French may be of great interest and usefulness for people working with the elderly population, whether in a research or clinical context.

## Methods

### Participants

The sample was composed of 238 French-speaking older people living in Belgium. The total sample was composed of people having participated in different surveys including the measure of interest (AAQ) conducted by the Psychology of Aging Unit (University of Liège). People were recruited from various social groups and organizations for retired seniors in Belgium (e.g., University of the Third Age and sports clubs) and by word-of-mouth. Inclusion criteria were age ≥ 60 and ability to read and write. People who reported severe visual or hearing impairment, severe health problems, dementia or other significant cognitive problems were excluded.

### Data Collection

In 87% of cases (*n* = 208), data were collected by a personal interview conducted at the participants’ residence or at the Psychology of Aging Unit. In 13% of the cases (*n* = 30), however, participants completed the questionnaire alone. Their scores did not significantly differ from those of people having completed the questionnaire during an interview.^1^ All participants completed the AAQ (*N* = 238). Test-retest reliability of the measure of interest was computed for 18 participants with a three-week interval. In addition, scores on subjective age (*n* = 101), quality of life (WHOQOL-OLD; *n* = 31), physical/mental health and depressed mood (*n* = 134) were used to assess convergent validity. Social desirability (DS-36) was also measured in 104 individuals. From one survey to another, the order of presentation of the AAQ was not the same. It was often completed at the beginning but could also be administered after other questionnaires assessing related concepts such as quality of life and health.

### Measures

**Demographic characteristics.** We assessed chronological age, sex, residence, marital status and educational level.

**Attitudes to Aging Questionnaire (AAQ).** This questionnaire (Laidlaw et al., 2007) assessed older adults’ attitudes toward their own aging process. It contains 24 items, divided into three subscales containing 8 items each: Physical Change, Psychological Growth and Psychosocial Loss. Respondents were asked to indicate their degree of agreement on a five-point Likert scale, where 1 reflects *strongly disagree* or *not at all true*, and 5 reflects *strongly agree* or *extremely true*. Scores range from 8 to 40 for each subscale. Higher scores on the Physical Change and Psychological Growth subscales indicate a more positive appraisal of one’s own aging. In contrast, higher scores on the Psychosocial Loss subscale indicate a more negative appraisal of aging. It is also possible to obtain a total score based on the total of all 24 items of the scale (summation of the inverted scores for the Psychosocial Loss subscale). Higher total scores reflect more positive attitudes toward one’s own aging process.

The questionnaire was translated and adapted from English into French (see Appendix 1) by three French speakers who were specialized in aging psychology and had good knowledge of English. A back-translation approach was not used because this method shows some limitations (McKenna & Doward, 2005; Swaine-Verdier, Doward, Hagell, Thorsen, & McKenna, 2004) and particularly, it is difficult to obtain back-translations identical to the forward translation. To ensure that the translated items were syntactically and semantically appropriate, differences between the translations were discussed and resolved by joint agreement of translators.

**Subjective age.** Participants were asked to specify orally, in years, how old they feel. We calculated the discrepancy between subjective age and chronological age as the dependent variable (i.e., discrepancy = subjective age – chronological age). A positive value indicates an older subjective age, and a negative value indicates a youthful subjective age. As mentioned, feeling older or younger is another expression of subjective aging.

**WHOQOL-OLD.** The French version of the WHOQOL-OLD (Leplège et al., 2013) is a questionnaire specifically developed to assess quality of life in older people (≥ 60 years). It consists of 24 items rated on a five-point Likert scale and covers six facets: sensory abilities; autonomy; past, present, and future activities; social participation; death and dying; and intimacy. Scores range from 4 to 20 for each subscale and from 24 to 120 for the total score, with higher scores corresponding to greater quality of life.

**Self-reported health and depressed mood.** Subjective mental and physical health was both assessed by a single item: “When you think of people of your age, how do you feel mentally/physically healthy compared to them?” with a Likert-type answering scale ranging from 1 (*not at all*) to 7 (*completely*). Depressed mood was assessed by the following question: “Do you think you have a depressed mood at the moment?” with a *yes/no* answer. If people answered positively, they had to precisely indicate the intensity of their negative feelings on a Likert scale ranging from 1 (very little) to 5 (very much). Since only 22 of the 134 participants gave an affirmative answer, a no answer was rated as 1 and a yes answer was rated as 2.

**Social Desirability Scale (DS-36).** The DS-36 (Tournois, Mesnil, & Kop, 2000) is a 36-item French questionnaire designed to assess two facets of social desirability: (1) auto-deception, that is, the tendency to give favourable but honest self-descriptions, and (2) hetero-deception, that is, the tendency to give an excessively favourable self-description to others. All items are scored on a Likert scale ranging from 0 (*totally false*) to 6 (*totally true*). The scores of each subscale theoretically range from 18 to 126. Higher scores indicate greater tendency toward auto- and hetero-deception.

## Analyses

Statistical data analyses were performed using Statistica 12 (Statsoft) and SPSS 23 (IBM Corp) and *p* < 0.05 was used as a criterion for statistical significance.

Data analyses were conducted in three parts. In the first part, we used an exploratory factor analysis to examine the factor structure of the AAQ. In the second part, we examined the scale’s acceptability and reliability. Finally, we assessed discriminant, convergent and known-groups validity and social desirability bias of the AAQ in subgroups of participants. Because some analyses were computed on a small sample (*n* < 50) or on ordinal variables (e.g., health), non-parametric statistics were used for all reliability and validity analyses and examination of social desirability bias. Parametric statistics were computed where applicable (i.e., for variables with normal distribution). Nevertheless, for more consistency and because results in parametric and non-parametric analyses did not considerably differ, results of non-parametric tests are presented.

To examine the factor structure of the data, the Kaiser-Meyer-Olkin statistic (KMO) and the Bartlett test of sphericity were used to test if there was an underlying structure to the data; KMO > 0.50 was required, as well as a significant Bartlett test. Principal component analysis with a direct oblimin rotation was then realized for the construct validity analysis. We preferred this oblique rotation to an orthogonal rotation (e.g., varimax) because Laidlaw et al. (2007) suggested a higher order factor in their confirmatory factor analyses. Therefore, we expected that variables being analysed represented a larger general concept (i.e., attitudes toward own aging) and could be interrelated. Factor loadings ≥ 0.40 were selected as a criterion to define a salient factor loading (Nunnally & Bernstein, 1994). The number of factors retained was decided using the following criteria: (1) Kaiser’s criterion of retaining factors with unrotated eigenvalues of approximately 1 or greater (Kaiser, 1960), (2) the scree test (Cattell, 1966), (3) Monte Carlo parallel analysis^2^ (Horn, 1965) and (4) the interpretability of the resulting factor structure (Gorsuch, 1983).

Acceptability was determined by examining AAQ missing values, summary score distributions, floor and ceiling effects, as well as skewness and kurtosis statistics. Floor and ceiling effects were present if more than 10% of respondents achieved the lowest or the highest possible summary score (Perales, Cosco, Stephan, Haro, & Brayne, 2013). Evidence of skewness or kurtosis were demonstrated if the absolute value of the statistic was higher than twice the standard error of the corresponding statistic.

Two types of reliability were then examined: internal consistency (Cronbach’s coefficients) and scale test–retest reproducibility. Values of 0.70 for Cronbach’s coefficients (DeVellis, 2011) and 0.20 for corrected item-total correlations (Streiner & Norman, 2008) indicate acceptable internal consistency and item homogeneity. Each of the subscales and the total score were assessed separately for reliability. Test-retest reliability, with a three-week interval between test and retest, was examined for a subgroup consisting of 18 participants. Spearman correlation coefficients and Wilcoxon signed-rank tests were used to test for temporal stability. Test–retest reliability correlations for summary scores ≥ 0.70 (Perales et al., 2013) and non-significant results for signed-rank tests (*p* > 0.05) indicate good reproducibility.^3^

To assess convergent validity, Spearman correlation coefficients were computed between the AAQ and scales assessing subjective aging or similar constructs. Convergent validity was assessed by examining association between the AAQ and subjective age (*n* = 101), the WHOQOL-OLD facets (*n* = 31), health and depressed mood (*n* = 134). Because a younger subjective age and positive attitudes toward one’s own aging are supposed to be different expressions of the same construct (i.e., subjective aging; Diehl et al., 2014), difference between subjective age and chronological age was assumed to be negatively correlated with positive attitudes toward one’s own aging. Based on previous validation studies (Chachamovich et al., 2008; Kalfoss et al., 2010; Lucas-Carrasco et al., 2013), it was hypothesized that AAQ Physical Change and Psychological Growth would positively correlate with the WHOQOL-OLD facets and subjective health and negatively with depressed mood. On the contrary, negative correlations were expected between Psychosocial Loss and the WHOQOL-OLD and subjective health.

Discriminant validity was assessed by computing sex-based differences with a Mann-Whitney U test; attitudes toward one’s own aging were expected to be unrelated to the sex of participants according to previous studies (Lucas-Carrasco et al., 2013; Top & Dikmetas, 2012; Top et al., 2012a).

Known-groups validity was assessed on the total sample by comparing scores for subgroups who are expected to have different attitudes toward one’s own aging. We examined differences between educational levels, as done by Lucas-Carrasco et al. (2013) and expected that higher educated people would have more positive attitudes toward their own aging. Mann-Whitney U tests were used to test for known group differences.

Social desirability bias was also tested by calculating Spearman correlation coefficients between the AAQ and DS-36 scales (*n* = 104). Because of the explicit nature of the items, we can expect that higher scores on the AAQ will be linked to higher social desirability scores: people with a tendency to perceive themselves more favourably and to give excessively favourable self-description to others (Tournois et al., 2000) will report more positive attitudes toward their own aging. Moreover, since some participants completed the AAQ and the DS-36 scales alone (*n* = 30) and others during a personal interview (*n* = 74), correlations between social desirability and the total score of the AAQ were also computed in the two groups to verify whether the context influenced these results.

## Results

### Sample characteristics

Participants were aged from 60 to 98 years old. The majority were women (66.4%) and 79.2% lived in their own homes (for more details, see Table 1).

**Table 1:** Demographic characteristics.

Sample characteristics (*N* = 238)	

**Age:** Mean (*SD*)	73.84 (8.66)
**Sex:*** n* (%)	
Male	80 (33.6)
Female	158 (66.4)
**Residence:*** n* (%)	
Home	190 (79.2)
Nursing home	50 (20.8)
**Marital Status:*** n* (%)	
Single	14 (5.9)
Married	97 (40.7)
Separated	40 (16.8)
Widowed	87 (36.6)
**Educational Level:*** n* (%)	
Primary & lower	91 (38.2)
Secondary	45 (18.9)
Higher	102 (42.9)

*Note. SD* = Standard Deviation.

### Factor structure

The KMO was greater than 0.50 (KMO = 0.78) and the Bartlett test of sphericity was significant (*p* < 0.001), indicating an underlying structure in the scale.

Eight factors with eigenvalues greater than 1 emerged, but the scree test and the parallel analysis suggested a maximum of four or three factors explaining, respectively, 42.75% and 36.90% of the total variance. Both a four- and three factor-factor analyses were inspected following oblique rotation; the four-factor solution produced one trivial factor with only three items loading ≥ 0.40 on this factor. The three-factor solution was then chosen: the first factor corresponded to the Physical Change scale, the second factor to Psychological Growth, and the third factor to Psychosocial Loss. The interfactor correlation between Factors 1 and 2 was 0.18, for Factors 2 and 3 was –0.10, and for Factors 1 and 3 was –0.24.

In the pattern matrix produced by the three-factor solution (see Table 2), all items loaded ≥ 0.40 on any factor, except for items 4, 8, 11, 13 and 19 (and among these, four loaded ≥ 0.35). Items 11 and 13, loaded more on the Psychological Growth subscale than on the Physical Change subscale and this was particularly notable for item 11 (≥ 0.35).

**Table 2:** Rotated factor matrix of the French AAQ : Factor loadings* (*N* = 238).

Items	Factor 1 Physical Change	Factor 2 Psychological Growth	Factor 3 Psychosocial Loss

1. As people get older they are better able to cope with life	–0.209	**0.578**	v0.050
2. It’s a privilege to grow old	0.029	**0.418**	–0.334
3. Wisdom comes with age	0.094	**0.386**	0.295
4. There are many pleasant things about growing older	0.023	**0.603**	–0.103
5. I am more accepting of myself as I have grown older	0.253	**0.453**	0.057
6. It is important to pass on the benefits of my experience to younger people	0.039	**0.581**	0.040
7. I believe my life has made a difference	–0.021	**0.361**	–0.254
8. I want to give a good example to younger people	–0.002	**0.497**	0.071
9. Old age is a time of loneliness	0.159	–0.008	**0.752**
10. Old age is a depressing time of life	–0.161	0.255	**0.491**
11. I find it more difficult to talk about my feelings as I get older	–0.296	0.199	**0.412**
12. I see old age mainly as a time of loss	0.058	–0.113	**0.617**
13. I am losing my physical independence as I get older	–0.386	0.184	**0.514**
14. As I get older I find it more difficult to make new friends	0.049	–0.139	**0.596**
15. I don’t feel involved in society now that I am older	–0.085	0.002	**0.547**
16. I feel excluded from things because of my age	–0.070	–0.214	**0.532**
17. It is important to take exercise at any age	**0.641**	–0.037	0.248
18. Growing older has been easier than I thought	**0.357**	0.322	–0.136
19. I don’t feel old	0.271	**0.399**	–0.135
20. My identity is not defined by my age	0.190	**0.231**	–0.049
21. I have more energy now than I expected for my age	**0.737**	0.157	0.044
22. Problems with my physical health do not hold me back from doing what I want to do	**0.537**	0.128	–0.167
23. My health is better than I expected for my age	**0.735**	0.007	–0.124
24. I keep myself as fit and active as possible by exercising	**0.752**	–0.056	–0.066

*Note*. *Items with the highest loadings within each factor are in bold type.

### Acceptability

As shown in Table 3, there were no missing values for any of the subscales. A non-normal distribution was observed for all dimensions, except the total score. Physical Change and Psychological Growth were negatively skewed and Psychosocial Loss had a flattened distribution. No noteworthy floor or ceiling effects were found per subscale or for the total score.

**Table 3:** Descriptive statistics and reliability parameters of the AAQ subscales (*N* = 238).

Subscales	Mean (*SD*) [Range]	Missing %	Floor/Ceiling %	Lilliefors normality test p-values	Skewness (*SD*)	Kurtosis (*SD*)	α

Total	85.67 (11.44) [56–117]	0.0	0.0/0.0	0.063	–0.06 (0.16)	–0.04 (0.31)	0.81
Physical Change	30.59 (5.06) [17–40]	0.0	0.0/3.4	< 0.001	–0.31 (0.16)	–0.17 (0.31)	0.75
Psychological Growth	28.95 (4.22) [17–40]	0.0	0.0/0.4	< 0.001	–0.33 (0.16)	0.13 (0.31)	0.62
Psychosocial Loss	21.87 (5.98) [8–39]	0.0	0.8/0.0	0.015	0.12 (0.16)	–0.51 (0.31)	0.74

*Note. SD* = Standard Deviation.

### Internal consistency

Internal consistency measured by Cronbach’s alpha was .81 for the total score, 0.75 for Physical Change, 0.62 for Psychological Growth and 0.74 for Psychosocial Loss (see Table 3). Taking into account the results of the factor analysis, Cronbach’s alpha of the Physical Change subscale without items 11 and 13 – loading more on the Psychological Growth subscale - was computed. An alpha of 0.76 was obtained (rather than 0.75) and Cronbach’s alpha of the Psychological Growth subscale with these items increased from 0.62 to 0.65. In addition, we noted that corrected item-total correlations were all above the threshold of 0.20.

### Test-retest reliability

Test–retest correlations were high for the total score (*r* = 0.79; *p* < 0.001) and all subscales (0.77 for Psychological Growth, 0.81 for Physical Change and 0.76 for Psychosocial Loss; all *p* < 0.001). Wilcoxon signed-rank tests showed no significant differences between test and retest (see Table 4).

**Table 4:** Test-retest reliability of the AAQ (*n* = 18).

Subscales	Test Mean (*SD*)	Retest Mean (*SD*)	*df*	*p*

Total	90.22 (11.23)	92.11 (13.30)	15	0.30
Physical Change	31.11 (4.83)	32.28 (5.42)	13	0.11
Psychological Growth	29.5 (4.59)	30.28 (4.79)	16	0.37
Psychosocial Loss	18.39 (4.74)	18.44 (5.55)	15	0.92

*Note. SD* = Standard Deviation. P-values for Wilcoxon signed-rank tests.

### Convergent validity

All correlations between the AAQ and other questionnaires are shown in Table 5. As expected, examination of total scores indicated that the more people were positive about aging, the younger they felt compared to their chronological age (*r* = –0.34, *p* < 0.001) and the more they reported greater quality of life (*r* = 0.51, *p* < 0.01). Similarly, the people with more positive attitudes toward their own aging reported more satisfaction with their subjective mental (*r* = 0.30, *p* < 0.001) and physical health (*r* = 0.46, *p* < 0.001). Those who reported not being depressed also had more positive attitudes toward their own aging (*r* = –0.40, *p* < 0.001).

**Table 5:** Convergent validity of the AAQ: Spearman correlations.

		Total	Physical Change	Psychological Growth	Psychosocial Loss

Subjective age	*n* = 101	–0.34***	–0.35***	–0.24*	0.20*
WHOQ-Tot	*n* = 31	0.51**	0.36*	0.28^b^	–0.37*
WHOQ-SAB	*n* = 31	0.18^a^	0.37*	–0.11^a^	–0.27^b^
WHOQ-AUT	*n* = 31	0.35^c^	0.30^a^	0.19^a^	–0.29^b^
WHOQ-PPF	*n* = 31	0.58***	0.33^c^	0.50**	–0.40*
WHOQ-SOP	*n* = 31	0.37*	0.20^a^	0.24^a^	–0.19^a^
WHOQ-DAD	*n* = 31	0.13^a^	0.03^a^	0.04^a^	–0.14^a^
WHOQ-INT	*n* = 31	0.20^a^	–0.00^a^	0.21^a^	–0.03^a^
Physical health	*n* = 134	0.46***	0.49***	0.06^a^	–0.40***
Mental health	*n* = 134	0.30***	0.24**	0.03^a^	–0.35***
Depressed mood	*n* = 134	–0.40***	–0.29***	–0.15^c^	0.37***

*Note*. Tot = Total; SAB = sensory ability; AUT = autonomy; PPF = past, present and future activities; SOP = social participation; DAD = death and dying; INT = intimacy. ^a^Non significant *p*-value, ^b^*p* < 0.15. ^c^*p* < 0.10. *p < 0.05. **p < 0.01. ***p < 0.001.

By examining the subscales more precisely, we observed that the more people had a positive view of their physical functioning (Physical Change), the less they reported the negative impacts of sensory losses on their quality of life (WHOQ-SAB; *r* = 0.37, *p* < 0.05) and the younger they felt (*r* = –0.35, *p* < 0.001), reporting a better mental (*r* = 0.24, *p* < 0.01) and physical health (*r* = 0.49, *p* < 0.001). In addition, people who experienced more psychological and social losses (Psychosocial Losses), were less satisfied with past and future activities (WHOQ-PPF; *r* = –0.40, *p* < 0.05), reported a worse physical (*r* = –0.40, *p* < 0.001) and mental health (*r* = –0.35, *p* < 0.001) and also felt older (*r* = 0.20, *p* < 0.05). People who reported a depressed mood also experienced more psychosocial losses (*r* = 0.37, *p* < 0.001). We also found that the more people perceived aging as a time of wisdom and gains (Psychological Growth), the more they were satisfied with past and future experiences (WHOQ-PPF; *r* = 0.50, *p* < 0.01).

### Discriminant validity

Analyses (see Table 6) showed no difference between men and women for the total score and the three AAQ’s subscales (all *p* > 0.05).

**Table 6:** Discriminant and known-groups validity of the AAQ.

	Total		Physical Change		Psychological Growth		Psychosocial Loss	

	Mean (*SD*)	*p*	Mean (*SD*)	*p*	Mean (*SD*)	*p*	Mean (*SD*)	*p*

Discriminant validity (*df* = 236)								
**Sex**								
Male (*n* = 80)	86.73 (10.25)		31.15 (4.76)		29.25 (3.40)		21.68 (5.86)	
Female (*n* = 158)	85.13 (12)	*0.51*	30.30 (5.19)	*0.30*	28.8 (4.40)	*0.36*	21.97 (6.06)	*0.86*
								
Known-groups validity (*df* = 236)								
**Living**								
At home (*n* = 60)	84.88 (9.86)		30.33 (5.35)		29.4 (3.70)		22.85 (4.98)	
Nursing home (*n* = 45)	78.47 (11.75)	*<0.01*	28.38 (5.92)	*0.04*	28.56 (4.46)	*0.46*	26.47 (5.67)	*<0.001*
**Education level**								
Primary & lower (*n* = 91)	83.15 (11.05)		29.73 (4.94)		28.74 (3.90)		23.31 (6.12)	
Secondary & higher (*n* = 147)	87.22 (11.44)	*<0.01*	31.12 (5.07)	*0.02*	29.09 (4.42)	*0.48*	20.99 (5.73)	*<0.01*

*Note. SD* = Standard Deviation. P-values for Mann-Whitney U tests.

### Known-groups validity

Results indicated (see Table 6) that participants with higher educational levels scored higher on the total score (*p* < 0.01) and Physical Change (*p* = 0.02) but lower on the Psychosocial Loss subscale (*p* < 0.01).

### Social desirability

The more people have positive attitudes toward their own aging (total score of the AAQ), the more they tend to give favourable but honest self-descriptions (*r* = 0.38, *p* < 0.001) or an excessively favourable self-description to others (*r* = 0.24, *p* = 0.01). Psychological Growth was also significantly correlated with both auto- (*r* = 0.32, *p* < 0.01) and hetero-deception (*r* = 0.35, *p* < 0.001). Psychosocial Loss and Physical Change subscale were significantly correlated with auto-deception (*r* = -0.31, *p* < 0.01 and *r* = 0.20, *p* = 0.01) but not with hetero-deception (*r* = –0.15, *p* = 0.13 and *r* = 0.10, *p* = 0.32).

When considering people completing the questionnaires alone, we also observed that attitudes toward one’s own aging (total score of the AAQ) are significantly correlated with both auto- (*r* = 0.47, *p* < 0.01) and hetero-deception (*r* = 0.57, *p* < 0.001). However, when data were collected during a personal interview, only auto-deception was significantly correlated with attitudes (*r* = 0.31, *p* < 0.01, but not with hetero-deception: *r* = 0.08, *p* = 0.53).

## Discussion

The aim of this study was to investigate the reliability and validity of the French adaptation of the AAQ in older people. This scale, aimed at assessing attitudes toward the aging process as a personal experience, gives priority to the subjective experience of aging rather than the objective indicators of physical or psychological health outcomes.

First, we noticed that no differences emerged among participants, whether the questionnaire was completed during a personal interview or alone. This result suggests that the AAQ is a good self-reported measure and can be used with large samples without requiring the presence of an interviewer (e.g., online surveys). Further, the structure of this French version was similar to the original one (Laidlaw et al., 2007) and scale’s acceptability was satisfactory. Moreover, the questionnaire demonstrated good test-retest reliability and internal consistency.

Concerning the factor structure, the Physical Change subscale was less conceptually homogenous. In fact, the distribution of items, with two exceptions, showed remarkable congruence with the factor solution reported by Laidlaw et al. (Laidlaw et al., 2007). The two exceptions were the items “I don’t feel old” and “My identity is not defined by my age”, which previously loaded on the Physical Change factor and now loaded on the Psychological Growth factor. Cronbach’s alpha values of these two scales did not change if these two items were placed in the Psychological Growth factor. Such results led us to choose the same structure as the original one, with three factors: Psychosocial Loss, Physical Change and Psychological Growth (Laidlaw et al., 2007).

Concerning acceptability, no floor or ceiling effects were observed for any of the subscales or the total score. This quality is important because floor and ceiling effects may limit a questionnaire’s ability to reveal changes (lower or higher scores) over time. With regard to internal consistency of the questionnaire used in the current study, the Cronbach’s alpha value of the total score (α = 0.81) was similar to the score obtained in Norwegian and Canadian samples (α = 0.86; Kalfoss et al., 2010). This suggests that items can be combined to form a summary score. Moreover, the Psychological Growth subscale demonstrated the lowest reliability, as observed in other validation studies (e.g., Lucas-Carrasco et al., 2013). In fact, even if item-total correlations indicated good consistency between items defining the respective scales, Cronbach’s coefficient of the Psychological Growth subscale was below the threshold of 0.70 (α = 0.62). It is possible that items of this scale are more sensitive to the translation procedure. This may also be inherent to the scale’s structure, utilizing both an experiential and general approach to aging. In fact, these two approaches may lead to different responses regarding items belonging to the same subscale. For example, respondents may have different opinions for general versus experiential items; indeed, they might answer general items by thinking about older people in general. In this case, it is possible that they have a more negative view of aging if they think about other older people in general rather than considering their own experiences and life. Particularly, the Psychological Growth scale contains four items using a general approach (against two items for the Psychosocial Loss and one for the Physical Change subscale). Therefore, the Psychological Growth domain results should be interpreted with caution.

In terms of convergent validity, we showed that positive attitudes toward one’s own aging are positively and significantly correlated with a younger subjective age, a finding that emerges for the total score as well as for the subscales. These results are consistent with the idea that attitudes toward one’s own aging and subjective age represent two expressions of subjective aging (Diehl et al., 2014). With regard to this result, we know that, even at advanced age, people are reluctant to identify themselves as being old (Kleinspehn-Ammerlahn et al., 2008). Moreover, experimental research revealed that a younger subjective age is a self-protective strategy displayed by older people in response to exposure to negative age-related information (Weiss & Freund, 2012; Weiss & Lang, 2012). A possible explanation of this observation might be that people don’t want to belong to a group negatively stereotyped and therefore, distance themselves from the negative stereotypes by feeling younger, which help them in maintaining a positive attitude toward their own aging. Nevertheless, it seems important to not neglect the possibility that older people who feel younger and exclude themselves from the old age category can endorse negative stereotypes about their own age group. According to Levy’s stereotype embodiment theory (2009), this might be problematic when people reach the age they consider as the “old age”.

Convergent validity was also supported by a strong positive correlation (*r* = 0.50) between the total score of the AAQ and the WHOQOL-total. Some subscales of these two questionnaires were also correlated with each other. Consistent with a previous validation study (Lucas-Carrasco et al., 2013), moderate correlations were found between the AAQ Physical Change and Psychosocial Loss subscales and the WHOQOL-OLD total. This suggests that the more people perceive their aging as a positive experience and have a positive view of their physical functioning, the greater their reported quality of life. Nevertheless, contrary to another validation study (Kalfoss et al., 2010) and recent cross-sectional studies (Top et al., 2012a, 2012b), we did not find significant correlations between most AAQ subscales and WHOQOL-OLD facets. This may be due to the smaller subsample (*n* = 31) used in our research compared to other studies. In fact, different correlations were not significant but inferior to the threshold of *p* = 0.15, which indicate significant trends. Interestingly, all these results showed associations between attitudes toward one’s own aging and quality of life in different cultures (Norwegian and English Canadian populations (Kalfoss et al., 2010), Spanish (Lucas-Carrasco et al., 2013), Belgian and Turkish (Top et al., 2012a, 2012b) populations. Moreover, an international study showed that attitudes toward one’s own aging assessed with the AAQ might mediate the relationship between health satisfaction and quality of life (Low, Molzahn, & Schopflocher, 2013). More precisely, this cross-sectional study of 4593 older people from 20 different countries showed that people who were dissatisfied with their health had more negative attitudes toward their own aging, which led them to report lower quality of life. In addition, we observed a positive correlation between positive attitudes toward one’s own aging and a good subjective physical health (*r* = 0.46). These results are consistent with a study showing that a negative view about aging influences the evolution of physical health (Levy, Slade, & Kasl, 2002), but it also suggests that current physical health may in turn influence attitudes toward one’s own aging and therefore, quality of life of older people. These results thus highlight the importance of taking attitudes toward one’s own aging into account.

Consistent with previous studies (Top & Dikmetas, 2012; Top et al., 2012a), we did not find differences between men and women on AAQ subscales and total score. Finally, the scale also showed a good known-groups validity. As reported by Lucas-Carrasco et al. (2013), higher educated people had a more positive view of their physical functioning during aging and reported less negative psychosocial losses. This result may reflect the possibility that higher educated people have more personal resources helping them to accommodate to age-related changes and maintain positive attitudes toward their own aging.

## Limitations

The present research has three notable limitations. First, it is difficult to ascertain that our sample is representative of the general older population. Indeed, even with diversified methods of recruitment (people were recruited in several clubs, associations, nursing homes, etc. and in different study settings), the more isolated people were probably not included. Moreover, the proportion of older people aged 65 years and more living in nursing homes in our sample is greater (20.8%) than the current proportion in Belgium (8%; Vander Stichele et al., 2006). Second, test-retest reliability, which was good, was computed on a sample of 18 older adults. However, these results need to be confirmed with larger samples.

Third, the scale in itself also presents some limitations. Since the scale uses both an experiential and general approach that may lead to different responses regarding items belonging to the same subscale, it seems that emphasizing the scale’s instructions is very important in order for older people to understand that their responses must reflect their attitudes toward their own aging. Moreover, we observed weak but significant correlations between the AAQ and a social desirability scale in the full sample. The more people gave favorable but honest self-descriptions (auto-deception) and also tended to describe themselves positively to others (hetero-deception), the more they reported positive attitudes toward their own aging. We also observed an influence of the context of participants’ responses on the relations between these constructs. More precisely, when people completed questionnaires alone, both auto-deception and hetero-deception showed a correlation with their attitudes, while only auto-deception was associated to attitudes when questionnaires were completed in person. Even if these results would be replicated on larger samples, we cannot exclude the possibility that social desirability bias and context of response have an impact on participants’ responses in the AAQ. It does not mean that the scale is not a useful tool and should not be used at all. It rather implies that use of this scale should be accompanied by some precautions aimed at controlling the social desirability bias (potential covariate) among respondents.

## Conclusion and implications

This study contributes to the existing literature aiming at applying the AAQ in cross-cultural contexts. Indeed, the French version of the AAQ presents a similar structure to the original questionnaire (Laidlaw et al., 2007). Moreover, the scale has acceptable psychometric qualities: its reliability and validity are satisfactory and even good. Particularly, the convergent validity was similar to results found in previous studies.

Since an instrument’s validation is a continuous procedure, the next step should be to confirm the factor structure of the AAQ with a new and larger sample from the same population. Particularly, it would be interesting to compare different models based on the results of this study (e.g., models including or not items with factor loadings lower than 0.40) and to verify if there is a second-order factor reflecting correlations among the three subscales. Multi-group confirmatory factor analyses should also be performed in order to test for measurement invariance across age groups, which is a necessary condition that needs to be fulfilled before this instrument can be used in longitudinal studies. Moreover, it would be interesting to examine the reliability and validity of the AAQ in different populations of older people (e.g., old people with a cancer).

As a self-reported measure, the AAQ is considered as a useful tool to assess attitudes toward one’s own aging in older people. Because it does not necessarily require the presence of an interviewer, this questionnaire can be used with large samples, which is particularly interesting in public health surveys. In line with longitudinal studies conducted by Levy’s and colleagues (Levy, 2009), such a questionnaire can add to the existing literature in studies focusing on predictive power and relationship between attitudes toward one’s own aging and health-related outcomes (e.g., evolution of a cancer) in French-speaking populations. A better understanding of the development of negative attitudes toward own aging may provide the foundation to develop educational and public health programs aimed at the improvement of attitudes related to own aging in the general adult population. Moreover, as already explained, it seems important to control for social desirability, whether the questionnaire is completed alone or in person. In this respect, it would be interesting to conduct a study investigating more precisely the influence of the responding context (e.g., alone or in person, paper-and-pencil or online survey) on the relationship between social desirability and attitudes toward one’s own aging. Such a study could bring helpful recommendations to improve the control of factors related to social desirability in self-reports of attitudes toward own aging.

In addition, because of its good test-retest reliability and acceptability, the AAQ may be useful to assess the impact of health and social care structures and services on the attitudes of older adults. In this regard, it is first important to consider the possibility that older adults with more negative attitudes toward their own aging might be less likely to take advantage of intervention programs because of lower self-efficacy expectations (Diehl et al., 2014). Older adults’ attitudes toward their own aging could therefore be considered as important intervention targets that could be evaluated regularly in clinical programs (Diehl et al., 2014).

It is also essential to acknowledge that attitudes of health care professionals directly influence older adults’ attitudes toward their own aging process, and therefore, impact upon their quality of life (Low et al., 2013) and physical health (e.g., Levy et al., 2009). Since education and training of health professionals may help to change attitudes toward the elderly, it may also influence attitudes of older people toward their own aging; the measure of this construct thus presents a particular interest in order to assess the effectiveness of such interventions. By way of illustration, health care professionals, such as those working in nursing homes, are particularly vulnerable to ageist stereotypes because they are constantly exposed to ill older people (“An elderly person is an individual with bad physical and/or mental health;” Kearney, Miller, Paul, & Smith, 2000). These ageist stereotypes lead nursing home caregivers to use “baby talk” or “elderspeak” (e.g., shorter sentences, slower, less complex and more repetitive speech) when they speak to residents, regardless of their physical or cognitive health (Kemper, 1994). This type of communication is problematic because it leads older people to feel less confident in their communication skills, therefore altering their view of themselves (Kemper, Othick, Gerhing, Gubarchuk, & Billington, 1998; Ryan, Giles, Bartolucci, & Henwood, 1986). In this context, it could be interesting to assess the effectiveness of training aiming at reducing ageist behaviors by considering older adults’ attitudes toward their own aging as indicators of changes among health care professionals in future interventions.

## Competing Interests

The authors declare that they have no competing interests.

## Footnotes

No differences emerged for the AAQ total score and subscales (*df* = 186; *p* > 0.15) between people living at home completing the questionnaire during an interview (*n* = 158) and those filling it alone (*n* = 30). Similar results are obtained if people completing the AAQ alone are compared to a random sample matched for age, sex and educational level (*df* = 58; *n* = 30).

Software retrieved from http://www.softpedia.com/get/Others/Home-Education/Monte-Carlo-PCA-for-Parallel-Analysis.shtml.

A Mann-Whitney U test was also used because the Spearman correlation coefficients may be higher than the true test-retest reliability; therefore, high coefficients (i.e., a linear function) do not necessarily mean that the results are stable over time. Indeed, subjects might have higher scores after the three-week interval, but if this raise is similar among them, the coefficient might be closed to 1, even if scores are not stable over time.

## Note from the authors

Pierre Missotten and Manon Marquet contributed equally to this work.

## Appendix 1

### French version of the AAQ

#### Attitudes to Aging Questionnaire (Laidlaw et al., 2007)

##### Version française : Marquet et al. Ce questionnaire vous demande comment vous ressentez le fait de vieillir

Veuillez répondre à toutes les questions. Si vous n’êtes pas certain de la réponse à donner à une question, veuillez choisir celle qui semble la plus appropriée. Ce sera souvent votre première réponse.

Gardez à l’esprit vos attentes, vos espoirs, vos passions et vos préoccupations. Nous vous demandons de réfléchir à votre vie en général.

Les questions suivantes demandent à quel point vous êtes d’accord ou non avec les énoncés. Si vous êtes d’accord avec ceux-ci, entourez la réponse « Tout à fait d’accord ». Si vous n’êtes pas du tout d’accord avec les énoncés, encerclez la réponse « Pas du tout d’accord ». Si vous voulez indiquer que votre réponse se trouve quelque part entre « Tout à fait d’accord » et « Pas du tout d’accord », entourez une proposition entre les deux.

**Table:**

	Pas du tout d’accord	Pas d’accord	Incertain	D’accord	Tout à fait d’accord

1. En vieillissant, les gens sont davantage aptes à faire face à la vie.	Pas du tout d’accord	Pas d’accord	Incertain	D’accord	Tout à fait d’accord
2. C'est un privilège de vivre vieux.	Pas du tout d’accord	Pas d’accord	Incertain	D’accord	Tout à fait d’accord
3. La vieillesse est une période de vie synonyme de solitude.	Pas du tout d’accord	Pas d’accord	Incertain	D’accord	Tout à fait d’accord
4. La sagesse vient avec l'âge.	Pas du tout d’accord	Pas d’accord	Incertain	D’accord	Tout à fait d’accord
5. Vieillir apporte beaucoup de choses agréables.	Pas du tout d’accord	Pas d’accord	Incertain	D’accord	Tout à fait d’accord
6. La vieillesse est une période de vie synonyme de dépression.	Pas du tout d’accord	Pas d’accord	Incertain	D’accord	Tout à fait d’accord
7. Il est important de faire de l’exercice physique à tout âge.	Pas du tout d’accord	Pas d’accord	Incertain	D’accord	Tout à fait d’accord

Les questions ci-dessous demandent à quel point pour vous, les énoncés suivants sont vrais ou pas. Si ceux-ci sont extrêmement vrais pour vous, entourez la réponse « Tout à fait vrai ». Si pour vous les énoncés ne sont pas vrais, encerclez la réponse « Pas du tout vrai ». Si vous voulez indiquer que votre réponse se trouve quelque part entre « Tout à fait vrai » et « Pas du tout vrai », entourez une proposition entre les deux.

**Table:**

	Pas du tout vrai	Pas tellement vrai	Moyennement vrai	Plutôt vrai	Tout à fait vrai

8. Vieillir a été plus facile que ce que je pensais.	Pas du tout vrai	Pas tellement vrai	Moyennement vrai	Plutôt vrai	Tout à fait vrai
9. Je trouve qu’il est plus difficile de parler de mes sentiments en vieillissant.	Pas du tout vrai	Pas tellement vrai	Moyennement vrai	Plutôt vrai	Tout à fait vrai
10. Avec l'âge, je m'accepte mieux.	Pas du tout vrai	Pas tellement vrai	Moyennement vrai	Plutôt vrai	Tout à fait vrai
11. Je ne me sens pas vieux.	Pas du tout vrai	Pas tellement vrai	Moyennement vrai	Plutôt vrai	Tout à fait vrai
12. Je perçois la vieillesse principalement comme une période associée à la perte.	Pas du tout vrai	Pas tellement vrai	Moyennement vrai	Plutôt vrai	Tout à fait vrai
13. Mon identité ne se définit pas en fonction de mon âge.	Pas du tout vrai	Pas tellement vrai	Moyennement vrai	Plutôt vrai	Tout à fait vrai
14. J'ai plus d'énergie que ce que j'espérais pour mon âge.	Pas du tout vrai	Pas tellement vrai	Moyennement vrai	Plutôt vrai	Tout à fait vrai
15. Je perds mon indépendance physique en vieillissant.	Pas du tout vrai	Pas tellement vrai	Moyennement vrai	Plutôt vrai	Tout à fait vrai
16. Mes problèmes de santé physique ne m'empêchent pas de faire ce que je souhaite.	Pas du tout vrai	Pas tellement vrai	Moyennement vrai	Plutôt vrai	Tout à fait vrai
17. En vieillissant, je trouve qu’il est plus difficile de se faire de nouveaux amis.	Pas du tout vrai	Pas tellement vrai	Moyennement vrai	Plutôt vrai	Tout à fait vrai
18. C'est très important pour moi de transmettre les bénéfices de mes expériences aux plus jeunes.	Pas du tout vrai	Pas tellement vrai	Moyennement vrai	Plutôt vrai	Tout à fait vrai
19. Je pense que ma vie a changé quelque chose.	Pas du tout vrai	Pas tellement vrai	Moyennement vrai	Plutôt vrai	Tout à fait vrai
20. Je ne me sens plus aussi impliqué dans la société, maintenant que je suis plus âgé.	Pas du tout vrai	Pas tellement vrai	Moyennement vrai	Plutôt vrai	Tout à fait vrai
21. J'aimerais donner un bon exemple aux plus jeunes.	Pas du tout vrai	Pas tellement vrai	Moyennement vrai	Plutôt vrai	Tout à fait vrai
22. Je me sens exclus de certaines choses à cause de mon âge.	Pas du tout vrai	Pas tellement vrai	Moyennement vrai	Plutôt vrai	Tout à fait vrai
23. Ma santé est meilleure que ce que j'espérais pour mon âge.	Pas du tout vrai	Pas tellement vrai	Moyennement vrai	Plutôt vrai	Tout à fait vrai
24. Grâce à l'exercice physique, je reste actif et en forme.	Pas du tout vrai	Pas tellement vrai	Moyennement vrai	Plutôt vrai	Tout à fait vrai
